# Study protocol: BRInging the Diabetes prevention program to GEriatric Populations

**DOI:** 10.3389/fmed.2023.1144156

**Published:** 2023-05-18

**Authors:** Jeannette M. Beasley, Emily A. Johnston, Mary Ann Sevick, Melanie Jay, Erin S. Rogers, Hua Zhong, Sondra Zabar, Eric Goldberg, Joshua Chodosh

**Affiliations:** ^1^Department of Nutrition and Food Studies, New York University, New York, NY, United States; ^2^Department of Medicine, New York University Grossman School of Medicine, New York, NY, United States; ^3^Department of Population Health, Institute for Excellence in Health Equity, New York University, New York, NY, United States; ^4^VA New York Harbor Healthcare System, Medicine Service, New York, NY, United States

**Keywords:** diabetes prevention, older adults, nutrition, physical activity, lifestyle change, virtual, social support, implementation science

## Abstract

In the Diabetes Prevention Program (DPP) randomized, controlled clinical trial, participants who were  ≥ 60 years of age in the intensive lifestyle (diet and physical activity) intervention had a 71% reduction in incident diabetes over the 3-year trial. However, few of the 26.4 million American adults age ≥65 years with prediabetes are participating in the National DPP. The BRInging the Diabetes prevention program to GEriatric Populations (BRIDGE) randomized trial compares an in-person DPP program Tailored for Older AdulTs (DPP-TOAT) to a DPP-TOAT delivered via group virtual sessions (V-DPP-TOAT) in a randomized, controlled trial design (*N* = 230). Eligible patients are recruited through electronic health records (EHRs) and randomized to the DPP-TOAT or V-DPP-TOAT arm. The primary effectiveness outcome is 6-month weight loss and the primary implementation outcome is intervention session attendance with a non-inferiority design. Findings will inform best practices in the delivery of an evidence-based intervention.

## 1. Introduction

Over one-quarter (29.2%) of US adults aged 65 and older have Type 2 diabetes (i.e., 15.9 million people), and the Centers for Disease Control and Prevention (CDC) estimated that an additional 26.4 million older adults had prediabetes, defined as fasting plasma glucose values of 100 to 125 mg/dl or hemoglobin A1C values of 5.7 to 6.4%, in 2019 (1). Evidence-based diabetes prevention strategies, such as the National Diabetes Prevention Program (DPP), reduce the risk of developing diabetes but remain underutilized. In the original Diabetes Prevention Program (DPP) study, the diet and physical activity intervention conferred a 71% reduction in risk of type 2 diabetes for the participants who were ≥60 years of age (*n* = 648) after 3 years of follow-up (2). Since the seminal DPP was established as efficacious (2), the intervention has been implemented in hospital, community, work, and other settings.

Despite this, just 14.6% of rural counties and 48.4% of urban counties in the nation have a DPP site. Barriers to DPP participation exist for both individuals and healthcare systems. Individuals face barriers to access, including travel to 22 in-person sessions, as well as cost if they elect to utilize a commercial online program. Older adults, in particular, face barriers to care, including transportation, costs, time burden, and limited physical function/reserve. Healthcare systems face significant cost burdens for training DPP facilitators and hosting the program (3). In the few studies that have reported on costs of administering the DPP, there was a 5-fold difference in cost per participant across studies, with a virtual program costing less than on-site delivery (4, 5). In the BRInging the Diabetes prevention program to GEriatric Populations (BRIDGE) trial, we will address these barriers by generating a DPP Tailored for Older Adults (DPP-TOAT) and delivered via Virtual sessions (V-DPP-TOAT).

Technology use among older adults is increasing. Almost two-thirds (63%) of adults aged 66–75 years use the Internet to access health information, including 49% of adults aged 75 and older (6). Furthermore, 69% of adults ≥65 have a mobile phone, including 56% of adults ≥75 years of age. Web-based interventions have effectively increased health knowledge (7) and physical activity among older adults (8–10). The proportion of older adults enrolling in internet-based programs will grow with the aging of younger cohorts who are more accustomed to depending on technology to meet their health care and other needs.

Delivering a DPP through a videoconferencing platform may extend the program’s reach into the older adult community that may otherwise lack access (11). The NYU Langone Health catchment area includes more than 7.2 million people, of whom nearly 1 million individuals are aged 65 and older. An intervention using technology to provide remote training and individualized feedback increases the likelihood of reproducibility outside of the NYU Langone Health network and sustainability over time (12). Virtual interventions do not require brick-and-mortar facilities or centralized staff; these highly scalable interventions can be delivered from any location. However, a recent behavioral weight loss trial reported that in-clinic group visits, but not telephone group visits, resulted in statistically significant greater weight loss at 24  months compared with traditional in-clinic individual visits (13). Lessons learned from the implementation evaluation of the BRIDGE DPP will provide the opportunity for other healthcare systems to offer the program to eligible patients. The study aims are to compare the effectiveness and implementation of V-DPP-TOAT versus DPP-TOAT within a large healthcare system.

## 2. Methods

### 2.1. Study design and overview

**Figure 1:**
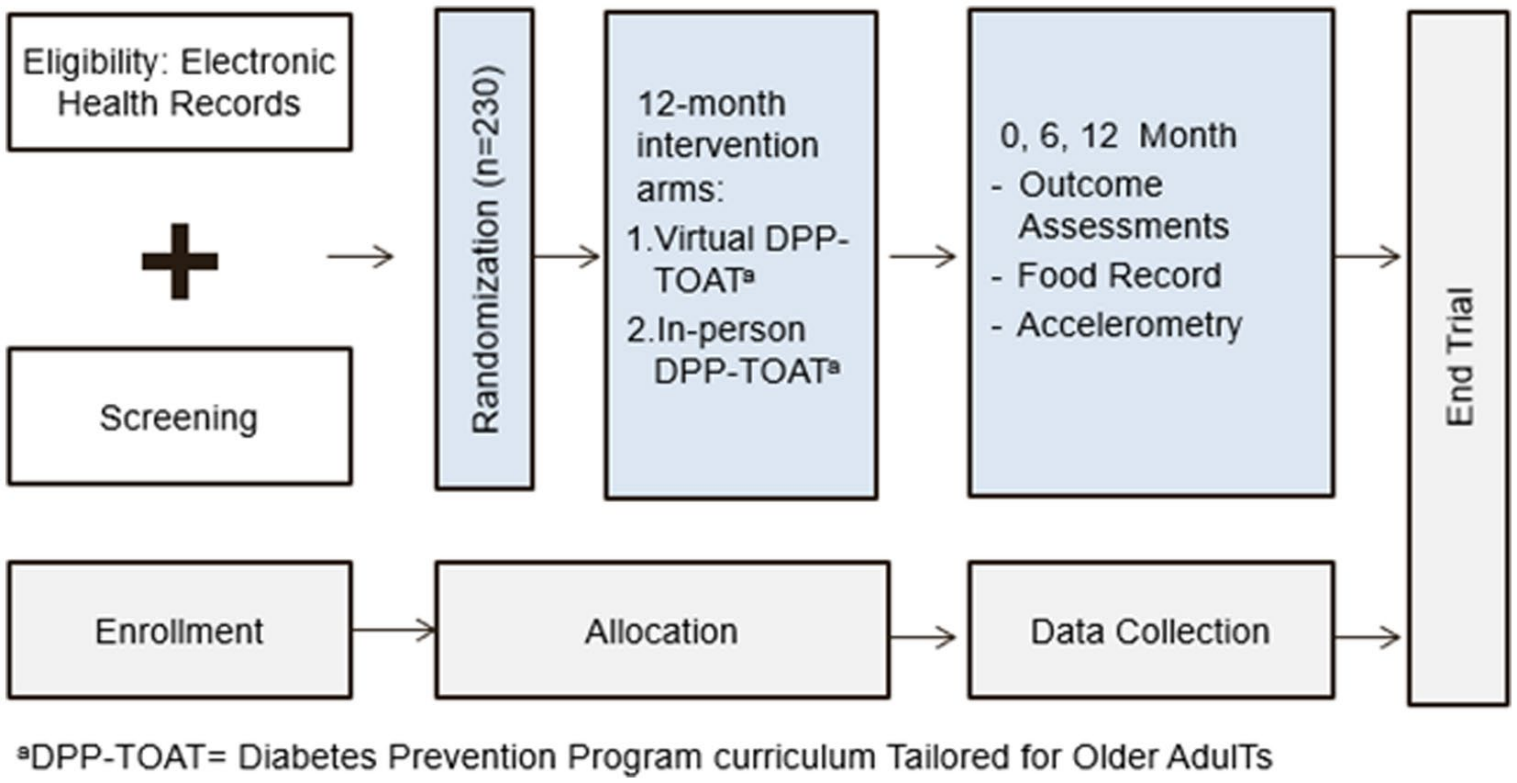
Study flow diagram.

We will conduct a type 1 hybrid (14), randomized, controlled trial of the BRInging the Diabetes prevention program to GEriatric Populations (BRIDGE) study, which will compare an in-person DPP Tailored for Older AdulTs (DPP-TOAT) to a DPP-TOAT delivered via group virtual sessions (V-DPP-TOAT; Figure 1). All randomized individuals (*N* = 230; 1:1 randomization) will receive 22 intervention sessions over the course of a year facilitated by a certified DPP lifestyle coach, but the delivery method will vary (virtual versus in-person).

Outcome assessments will be conducted at 0, 6, and 12 months at the clinic where we will assess weight, hemoglobin A1c, diet, and physical activity in both groups. At baseline, we will assess height, weight, waist circumference, and administer questionnaires to all participants. The trial design will adhere to the CONSORT checklist (see Appendix A), including an intention-to-treat analysis (15). Meticulous adherence to the study protocol and fidelity monitoring will ensure robust and unbiased results.

### 2.2. Recruitment

The NYU Langone Health patient population includes over 15,000 patients aged 65 or older with a diagnosis of prediabetes who meet the basic inclusion criteria. NYU Langone Health clinical providers utilize Epic, one of the country’s largest electronic health record platforms, and its patient portal (*MyChart*), which allows for direct outreach and bi-directional communication with patients. Using Epic, we will continuously identify eligible older adults with prediabetes. As in our prior work (16–19), patients will be recruited through both physician referral and proactive outreach to patients. For proactive outreach, we use Epic-generated lists of eligible patients based on our eligibility criteria. A research team member will send lists to patients’ primary care provider (PCP), who will subsequently identify any contraindications to participating. Potential participants will be sent a message through the patient portal (*MyChart*) and/or mailed a letter signed by the principal investigators (JB and JC) and medical director (EG) that describes the study and gives them the opportunity to opt out (i.e., request not to be contacted). We will then call potential participants to recruit, screen for eligibility, and schedule a baseline visit. Table 1 outlines eligibility criteria for study participation.

**Table 1:** Eligibility criteria.

Inclusion Criteria
• Aged ≥65 years*
• Prediabetes (A1C 5.7–6.4%, fasting glucose 100–125 mg/dl, or oral glucose tolerance test of 140–199 mg/dl within 12 mo) *
• BMI of ≥25 kg/m^2^, ≥ 23 kg/m^2^ if Asian American*
• English speaking*
• Under the care of PCP in the NYU Langone Health system*
• Able to travel to NYU medical center for in-person evaluations *
Exclusion Criteria
• Prevalent diabetes or End-Stage Renal Disease (> = CKD stage 3)*^±^
• Prior participation in Diabetes Prevention Program^±^
• A documented history of active psychosis or other cognitive issues via ICD-10 codes*
• Taking FDA-approved weight loss medications*^±^
• PCP stating that patient should not participate
• Inability to communicate due to severe, uncorrectable hearing loss or speech disorder^±^
• Severe visual impairment (if it precludes completion of assessments and/or intervention)^±^

*if assessed through EPIC, ±if assessed during screening. A1c, hemoglobin A1c; BMI, body mass index; mo, months; PCP, primary care provider; FDA, Food and Drug Administration.

#### 2.2.1. Randomization into Intervention Arms

The study statistician (HZ), will generate randomized treatment allocation in a blinded environment using a Research Electronic Data Capture (REDCap, https://projectredcap.org/) randomization tool.

#### 2.2.2. Retention

We will use the following strategies to encourage retention of participants in both arms of the study: (1) reminder of study visits and intervention sessions via email and phone call, (2) financial incentives following successful completion of study visits ($50 gift card for each baseline, 6, 12 month visits), (3) birthday cards, (4) certificates of completion and milestone emails.

#### 2.2.3. Masking

As with most behavioral trials, it is difficult to mask participants to the intervention arm. To reduce bias, all outcome assessors will be masked to the intervention arm and participants will be asked not to disclose treatment allocation. To detect potential unmasking, we will ask outcome assessors about participant allocation after each post-randomization measurement visit.

### 2.3. Intervention arms

#### 2.3.1. Common components to both interventions: V-DPP-TOAT and DPP-TOAT

Both programs will be composed of sixteen 60-min weekly/bi-weekly sessions followed by six 60-min monthly support sessions (see Appendix B) with a group size of 8–15 facilitated by a certified DPP lifestyle coach. The DPP intervention is based on Social Cognitive Theory (20), which focuses on the role played by self-referent thought in the maintenance of behavior change. The following describes the theoretical concepts threaded throughout the intervention sessions. Self-efficacy (e.g., the participant’s confidence in their ability to engage in healthier behavior) is derived from four major sources of information: (1) mastery experiences; (2) social modeling; (3) verbal persuasion; and (4) physiological states. Mastery experiences emphasize past successes; setting incremental, easily achievable goals; identifying modifiable barriers to healthy behavior; receiving positive feedback on goal achievement; and practicing problem solving skills around barriers to adherence. Social modeling enhances mastery when participants share their successes and help each other problem-solve around barriers they encounter. Verbal persuasion emphasizes the participant’s previous successes to demonstrate their capability (e.g., “As a result of your effort, you lost a pound last week. You can do it again.”) We will assist participants in recognizing physiologic benefits they experience from lifestyle change (e.g., more energy, better sleep, or BP control). Intervention materials, which are based on the 2021 PreventT2 lifestyle change program (21), will include:

- *Participant Manual*: An evidence-based manual describing detailed program content and personalized calorie and macronutrient recommendations to achieve the desired weight. Written at the sixth-grade reading level in a large (14 point) sans serif font, self-assessments and goal setting procedures will be included along with approaches to self-monitor food intake, physical activity, and cooking and meal pattern guides. To enhance self-identification with the program and for ongoing visual cues for program affiliation, we will provide tote bags inscribed with the institutional logo.
- *Tailoring of content for aging*: Based on our prior work (22), we have adapted group sessions to the unique needs of older adults. Resources include healthful eating strategies for dentition issues, changes in taste, and special attention to interactions between food and medications. Physical activity sessions provide adaptations for physical limitations, including chair exercises and strength training.
- *Food Guide*: Resources developed by the United States Department of Agriculture (23) providing tailored advice for older adults will be provided, along with instructions and tips for using programs such as Healthwatch 360 (24) to track calorie and nutrient intake compared to recommendations.
- *Hearing Assistance*: For participants in the in-person arm, we will offer a personal amplifier (PA; PockeTalker™) to all participants to address any self-reported or suspected hearing loss. A simple PA consists of an amplifier and microphone with the amplifier feeding a speaker’s voice directly into the wearer’s ears via headphones or ear buds. These devices reduce the difficulty with hearing in “difficult listening situations” by feeding the speaker’s amplified voice directly into the patient’s ear, while headphones or ear buds muffle external sounds (i.e., signal is louder than background noise level). These devices require no professional customization; simple volume and tuning controls allow for use directly out of the box. PAs enhance communication for those who have hearing loss in a variety of clinical settings (25–27). PAs were equivalent in performance to hearing aids in one randomized controlled trial (28), sound quality of PAs was preferred over hearing aids, improved physician-patient communication for elderly hospitalized patients (29) and understanding discharge information in an Emergency Department setting (30). Participants in the virtual arm will be offered ear buds to address any self-reported or suspected hearing loss.
- *Videos*: Each intervention session will also feature educational and behavioral coaching videos developed by our team or reputable organizations such as the National Institutes Health and VA MOVE (Weight Management Program, supported by VA’s National Center for Health Promotion and Disease Prevention) (31). The videos last 3–5 min, and will be used to anchor the discussion. Videos will also be available on the study website for participants to review as they desire.
- *Smart Scales*: We will provide participants with a Renpho® (Joicom Corporation, Eastvale, CA) Smart Scale for Body Weight, Digital Bathroom Scale for self-monitoring purposes and to monitor weight given the possibility of disruptions in in-person study visits due to COVID-19 pandemic restrictions. Renpho® Scales will transmit weights to researchers automatically using BlueTooth technology and have been used in other weight management studies. A research assistant will instruct each participant during the baseline visit on using the scale to report weekly weights, providing written instructions as well as a phone number and email address to obtain technical support as needed.

##### 2.3.1.1. V-DPP-TOAT Arm specific information

Intervention sessions will take place via videoconference rather than in-person, and we will encourage participants to use their preferred device to connect with the group (e.g., tablet, computer, and smartphone). Participants will be offered a tablet computer if they do not have an electronic device they’d like to use to connect. A research assistant will orient each participant to the tablet during the baseline visit, providing written instructions, a phone number and email address to obtain technical support as needed. Before or after the intervention session, participants will be assigned to breakout rooms where they will have a 1-on-1 visit with the lifestyle coach, report their weight, physical activity minutes and discuss action plan and goal achievement.

##### 2.3.1.2. DPP-TOAT Arm specific information

All interactions will be in-person and weights will be measured immediately before or after intervention sessions using a Renpho® scale.

### 2.4. Data collection

In-person data collection will occur during study visits for both arms. Assessments will occur at: baseline (first in-person encounter), six, and 12 months (Tables 2, 3). Participants will meet with a research assistant masked to treatment assignment to complete surveys and to measure glycemia, weight, height, and waist circumference (baseline, six, and 12 months). Research staff will administer all surveys, entering data directly into a REDCap database. The DPP facilitator will record DPP attendance data.

**Table 2:** Aim 1 BRIDGE measures.

Measures	Month			0	6	12
Demographics, health-related characteristics^1^^,^^2^^,^^3^	X		
Anthropometrics (height, weight, waist circumference)^4^	X	X	X
Hemoglobin A1c^5^	X	X	X
Social support^6^	X	X	X
Self-efficacy^7^	X	X	X
Dietary intake including alcohol, Food records (4 days including weekend day)	X	X	X
Physical activity record	X	X	X
Physical activity/sleep, accelerometry	X	X	X
Smoking^8^	X	X	X
Quality of life^9^^,^^10^	X	X	X
Physical function, walking limitations^11^	X	X	X

1 Prediabetes Risk Test (32).

2 Health literacy (33, 34).

3 Technology use (35).

4 Anthropometric measures (36).

5 Blood draw per standard HbA1c protocol.

6 NIH adult toolbox social relationship scales (37).

7 Weight efficacy lifestyle questionnaire (38).

8 Smoking (39).

9 Emotional wellbeing (40).

10 Summary score (40).

11 Physical functioning (40).

**Table 3:** Aim 2 BRIDGE measures.

		Month		
Component		0	6	12
R	Recruitment^1^^,^^2^^,^^3^	X		
E	Weight and HbA1c outcomes^4^	X	X	X
A	Adoption by the healthcare system^5^		X	X
	Fidelity to CDC DPP curriculum^6^		X	X
I	Participant attendance at group sessions^7^		X	X
	Participant and DPP facilitator acceptability^8^		X	X
	Cost analysis^9^			X
M	Weight and HbA1c outcomes^4^			X
	Program maintenance and growth^5^			X

RE-AIM, reach, efficacy, adoption, implementation, maintenance; HbA1c, hemoglobin A1c; CDC, Centers for Disease Control and Prevention; DPP, diabetes prevention program.

1 Use EPIC to evaluate the number (proportion) of individuals who are:

a Eligible for the study based on EHR data.

b Invited to participate through MyChart and/or via study recruitment mailings.

c Responsive to study invitation.

d Enrolled in study.

2 Evaluate factors associated with participation by comparing the demographic and clinical characteristics of patients reached by the intervention vs. those not reached.

3 Evaluate reasons for not participating (one multiple choice question at end of screening).

4 Outcomes assessment per standardized procedures (see aim 1 measures table).

5 Intervention process data and key informant interviews.

6 Training Period – Monitor skill acquisition and quality of counseling.

• Lifestyle coaches completed CDC training via New York City Department of Health.

• Curriculum certification requirements are built into data collection (e.g., attendance, weekly weigh-ins, physical activity minute tracking).

• Intervention Period – Record group sessions throughout the intervention period, and a study team member will monitor 10% of the sessions.

o Fidelity Checklist.

o Will provide refresher training if fidelity checklist ratings average < 80%.

7 Measure attendance, outcomes assessment completion, and diet and physical activity monitoring completion.

8 Acceptability and Feasibility.

9 Costs associated with staffing, supplies, and participant time for each arm.

#### 2.4.1. Aim 1: to evaluate the effectiveness of the V-DPP-TOAT compared to the DPP-TOAT

Core measures for Aim 1 are displayed in Table 2.

#### 2.4.2. Aim 1: quantitative data analysis

We will compare the effect of the V-DPP-TOAT versus the DPP-TOAT interventions on weight and glycemia, which are both continuous outcomes. First, we will report means and standard deviations by randomization assignment and assess the assumption of normality in the outcomes. We will estimate the model using ordinary least squares regression

$$Y6_{i}=\beta_{0}+\alpha_{0}Y0_{i}+\alpha_{1}P_{i}+e_{i}$$

where $Y6_{i}$ is the 6-month weight or Hemoglobin A1c for participant i . $Y0_{i}$ is the baseline weight or A1c for participant i . $P_{i}$ is an indicator variable, where $P_{i}=1$ if the participant i has been randomized to V-DPP-TOAT, and $P_{i}=0$ for the DPP-TOAT. $e_{i}$ is an error term. The parameters to be estimated are as follows: $\beta_{0}$ is the average for those in the DPP-TOAT group; $\alpha_{0}$ represents the relationship between the baseline and follow-up outcome (weight or A1c) measurement; if the relationship is strong, the inclusion of the baseline measurement can increase statistical power; and $\alpha_{1}$ represents the effect of the V-DPP-TOAT intervention. Specifically, the hypothesis test will compare $H_{0}:\alpha_{1}<\Delta$ vs. $H_{A}:\alpha_{1}\geq -\Delta$ with the noninferiority margin Δ set as 5% for each of the outcomes. We will calculate the 95% confidence interval of $\alpha_{1}$ . If the lower bound is above the margin -Δ, the V-DPP-TOAT is deemed non-inferior and the trial is a “success.” Further, if the lower bound of that same CI is also above zero, then superiority of V-DPP-TOAT can also be declared (41). Interaction terms between $Y0_{i}$ and $P_{i}$ will be tested to see if there is effect modification by any of the baseline covariates.

We will assess heterogeneity in outcomes among healthcare settings/clinics by estimating an intraclass correlation coefficient (ICC). If the heterogeneity cannot be ignored, we will use a multi-level model with nested random effects in the above equation to accommodate the correlations caused by healthcare settings/centers. Outcomes analysis will use an intention-to-treat principle, with a per-protocol analysis as a sensitivity analysis (41) employing R software (version 1.2.5019, R Foundation for Statistical Computing) for all analyses.

##### 2.4.2.1. Sensitivity analysis for missing data

We will compare demographic and clinical covariates in participants with and without missing data to identify factors potentially contributing to missingness. We will use 10 multiple imputations from the original dataset using the predictive mean matching method substituting missing values within each impute.

##### 2.4.2.2. Power: aim 1

To achieve 80% power to detect non-inferiority using a one-sided, two-sample *t*-test (lower bound), we will need 85 participants per group. The margin of non-inferiority is 5% of weight loss in the DPP-TOAT arm. The true efficacy difference between the 6-month weight loss is assumed to be 1.6 kg (weight loss of 5 kg in the V-DPP-TOAT arm and 6.6 kg in the in-person DPP-TOAT arm with standard deviation of 5 kg in each arm (42)). The significance level (alpha) of the test is 0.05. A total of 230 participants are needed, assuming 25% attrition at 6 months based on data from our work and others (22, 42, 43).

#### 2.4.3. Aim 2: to evaluate the implementation of DPP-TOAT

To determine the feasibility of generalizing the DPP-TOAT intervention to other clinics within the NYU Langone Health system in New York and, ultimately, to other states, we will evaluate the implementation of the program during the RCT. Based on previous work, we hypothesize that adherence to the V-DPP-TOAT will be greater than the DPP-TOAT, as measured by the number of group sessions completed by each participant (42). We will use the RE-AIM (reach, effectiveness, adoption, implementation, maintenance) framework (44) to assess quantitative implementation outcomes during the Aim 1 effectiveness trial (Table 3). We will use the Consolidated Framework for Implementation Research (CFIR) (45) to examine barriers/facilitators to implementation within five main domains (intervention characteristics, inner setting, outer setting, participant/stakeholder characteristics, and implementation process), ensuring that this intervention can be generalized and readily disseminated and implemented elsewhere.

- Reach: We define the reach of the intervention as the number (proportion) of individuals who are (a) eligible for the study based on electronic health record data, (b) exposed to recruitment, (c) who initially responded, and (d) who decided to participate. We will survey those who decline participation to evaluate reasons for not participating. We will evaluate factors associated with participation by comparing the demographic and clinical characteristics of patients reached by the intervention vs. those not reached.
- Effectiveness: We will explore potential outcome differences in subgroups (e.g., sex, BMI category). We will conduct descriptive exploratory data analyses to explore which components may contribute most to the improvement of the outcomes.
- Adoption: We will assess willingness to adopt the intervention if it is found to be effective among key settings serving older adults with prediabetes, including other healthcare systems (e.g., a public hospital setting, senior centers, and rural settings, identifying barriers/facilitators toward future adoption.
- Implementation: We will evaluate implementation fidelity and resource requirements. We will also evaluate the acceptability and feasibility of the intervention among participants and facilitators, as well as barriers to implementing the program during the trial and to inform future implementation efforts. We will collect data on time spent delivering the intervention by lifestyle coaches including number of sessions, cost of materials, and other program costs.
- Maintenance: We will assess long-term maintenance of primary clinical outcomes (weight maintenance and glycemia at 12 months post-baseline visit).

##### 2.4.3.1. Aim 2 data collection

Research assistants keep detailed recruitment records using standardized forms to capture variables related to intervention *reach* described above (e.g., percent eligible, percent who enroll). Recordings of each session provide rich data related to *implementation fidelity* described above (e.g., number of group sessions completed, session length, topics covered during each session). Core measures for Aim 2 are listed in Table 3.

##### 2.4.3.2. Quantitative data analysis

Aim 2 uses the RE-AIM framework to evaluate implementation of the V-DPP-TOAT and in-person DPP-TOAT interventions. We will compare the number of group sessions completed by each participant in the two arms using generalized linear models with count outcomes, because DPP session attendance is positively associated with weight loss (42, 46), and is a CDC recognition status benchmark (47). The proportion of participants who complete at least eight intervention sessions will be compared by logistic regression between the two arms to make our findings comparable to other DPP implementation studies (42, 46). However, our primary implementation outcome of attendance is independent of any threshold chosen in the number of sessions. We will test interaction terms between baseline covariates and treatment assignment to determine if there is effect modification by any of the baseline covariates.

If there are imbalances at baseline for any important participant health, demographic or other factors, we will adjust the models to take these imbalances into consideration. For the remaining analyses, we will conduct similar types of analyses, using logistic regression for categorical outcomes and linear regression models for continuous outcomes.

We will use mediation analysis to dissect the indirect effects of the treatment acting through the intermediate variables (social support, self-efficacy, and self-monitoring) on the primary outcome (attendance), and the direct effects of treatment on the outcome (attendance) not mediated through the mediators. For each of the three mediators, an overall score and subscale scores will be computed by summing relevant items. This analysis will help understand the underlying treatment mechanisms of behavior change. Specifically, we will use the principal stratification and structural mean models. We will assess the assumptions necessary for attaining identifiability of key parameters of the basic causal model and perform a series of sensitivity analyses. We will also investigate the interactions between baseline covariates and the mediation effects to determine whether any baseline factors modify the mediation effects (48, 49).

In order to inform cost for future implementations and policy makers (4), we will compare the sum of variable (e.g., operating costs, supply costs, percent time of intervention and staff) and fixed costs (e.g., space, equipment) between intervention arms (50).

##### 2.4.3.3. Aim 2 power

Based on data from Lee 2018, we expect a higher proportion of V-DPP-TOAT participants will complete at least eight intervention sessions compared to DPP-TOAT participants (89 vs. 63%, respectively) (42). With 90 samples in each arm at 6 months, we achieve 98% power to detect the difference in session completion proportions of 26% at alpha = 0.05 (89 vs. 63% respectively) and 80% power to detect smaller difference of 19% (82 vs. 63% respectively).

##### 2.4.3.4. Aim 2 qualitative data

All interviews will be recorded using Zoom (in-person sessions will use Zoom via conference room video and audio system) with transcripts saved. Data analysis from transcriptions will follow techniques of narrative analysis (51, 52) and a “constant comparison” analytic approach (53). To code transcripts, the research team will develop an initial set of codes, informed by the open-ended questions and the interviews’ guiding conceptual framework. For each core code, we will ultimately develop one or more “secondary codes” that represent either more specific or restricted aspects of the phenomenon for contextualization or to suggest underlying personal meanings. The secondary codes will vary in specificity or subtlety depending on the judged substantive value of additional refinements. The coding schema is a strategy for organizing and assimilating the large amount of data that the interviews will yield. To ensure that the coding is both valid (i.e., well grounded in the data) and reliable (consistent in meaning), the criteria for assigning a specific code to a block of text will be systematically developed and well documented. The resulting codebook will be refined and expanded upon to reflect and incorporate emerging insights throughout the coding process. All transcripts will be double-coded, and discrepancies will be resolved by consensus discussion.

The coded transcripts will be analyzed with *Atlas.ti* (Version 8, Berlin, Germany), a software package for qualitative data analysis. With *Atlas.ti*, concepts (constructs, themes), contextual factors, participant characteristics, behaviors and attitudes can be related to one another. This will be particularly important to examine, for example, how themes vary or are consistent across different participant subgroups. Frequency counts will be generated for core or secondary codes by identified subgroups, and the total data file.

##### 2.4.3.5. Intervention fidelity

We will use a fidelity checklist to monitor skill acquisition and quality of counseling. A study team member will monitor 10% of the recorded intervention sessions using a fidelity checklist. We will provide refresher training if fidelity checklist ratings average <80%. We will measure attendance, outcomes assessment completion, and diet and physical activity monitoring completion.

*Dissemination*. The RE-AIM and CFIR frameworks analyses will address generalizability and transportability to facilitate future dissemination.

## 3. Discussion

Previous DPP studies have shown great benefit for older adult participants with reductions in incidence of diabetes of up to 71% (2). Barriers to attendance in older adults limit participation and barriers to engagement may include reduced hearing acuity in a group setting, reduced visual acuity and ability to read DPP materials, and reduced applicability of diet and physical activity recommendations. These barriers may be attenuated by adaptations made in the BRIDGE study.

This work will contribute to the growing body of implementation studies that at present, consist largely of commercial online DPP programs that engaged older adults (Table 4) (42, 46, 54, 55). Most recently, Omada’s randomized, controlled trial recruiting from primary care centers in Nebraska demonstrated meaningful reductions in weight and HbA1c (42, 46, 54, 56–58). However, none of the commercial programs provide facilitated theory-based group sessions. Data from our work and others suggests the social support provided by these sessions is critical for engagement and retention of older adults (22, 43, 59). Furthermore, existing programs do not offer adaptations for older adults for nutrition, physical activity, hearing impairment, and/or low vision. Due to the COVID-19 pandemic, Medicare allowed for virtual delivery of the DPP (60), and it is likely that demand for convenient, online programs will continue (61, 62). The goal of this project is to test the effectiveness of a virtual adaptation that will: (1) be accessible to those who cannot afford to pay for commercial programs; (2) provide social support by the facilitated group sessions; (3) reduce the barriers associated with in-person DPP programs; and (4) serve the unique needs of older adults.

**Table 4:** Healthcare system implementations of online diabetes prevention programs (DPP) engaging older adults.

Lead Author/Year	Study design	N	Population	Program	Program delivery	Results
Block, 2015 (54, 55)	Randomized, controlled trial	339	Recruited from Palo Alto Medical Foundation; Mean age 55.0 (8.9)	Alive PD-1-year program of regular contact and goal setting, weekly in the first 6 months and biweekly thereafter, plus midweek automated email and mobile phone	Automated online platform with interactive voice response phone calls and a mobile app.	Alive-PD participants achieved significantly greater reductions than controls in fasting glucose (mean − 7.36 vs. −2.19 mg/dl, *p* < 0.001), HbA1c (mean − 0.26%vs. mean − 0.18% *p* < 0.001), and body w eight (mean − 3.3 vs. −1.3 kg, *p* < 0.001).
Castro Sweet, 2018 (46)	Single-arm, retrospective	501	Humana Medicare Advantage beneficiaries with evidence of prediabetes/metabolic syndrome	Prevent – a DPP-based group lifestyle intervention that integrates a private online social network, weekly lessons, health coaching, and a w ireless scale and pedometer. Consists of a core 16-w eek intensive lifestyle change intervention and post-core lifestyle change maintenance intervention	Online platform, mobile app	92% completed at least 9 of 16 core lessons. At 12 months, average weight loss was 7.5% (7.8). Among participants with clinical data, average HbA1c decrease was 0.14% (*p* = 0.001) and average total cholesterol decrease was 7.08 mg/dl (*p* = 0.008). Self-reported w ell-being, depression, and self-care improved (*p* < 0.0001).
Lee, 2018 (42)	*Post hoc* analysis of two prospective, pragmatic, nonrandomized studies	≥65 years: 120 <65 years: 258	Veterans with prediabetes	VA-DPP – DPP Group Lifestyle Balance curriculum’s 16 core sessions over 6 months followed by 6 monthly maintenance sessions Online-DPP – Prevent curriculum, weekly online modules over 12 months	VA-DPP: In-person Online-DPP: Online platform, mobile app	A higher proportion completed eight or more sessions in the Online-DPP intervention than in the VA-DPP intervention (*p* < 0.05). Veterans ≥65 years achieved similar participation and weight loss as younger adults, whether DPP was delivered in person or online. Both age groups lost a clinically and statistically significant amount of weight (5 kg or 5% weight at 6 and 12 months; *p* < 0.05) and had similar w eight loss trajectories over the 12 months (*p* < 0.05).
Almeida, 2020/Katula, 2020 (56, 57)	Randomized, controlled trial	599	Nebraska Medicine primary care clinics	Omada Health Program – 16-week intensive curriculum focusing on w eight loss follow ed. by 36-week curriculum focusing on weight maintenance	Online platform, mobile app versus one two hour group diabetes prevention class	Omada produced significantly greater reductions in HbA1c (−0.23 vs. −0.15%, *p* < 0.01) and reduction in percent change of body w eight from initial w eight (−5.4 vs. −2.0%, *p* < 0.01) relative to control at 12 months.
Fitzpatrick 2021 (55)	Mixed-methods natural experiment	7,123	Kaiser Permanente Northwest patients	In-person DPP Digital DPP versus Omada Health Program compared	In-person versus online platform	In progress

DPP, diabetes prevention program; HbA1c, hemoglobin A1c; VA, veterans administration.

Our study has several strengths. The results of our trial can directly inform policy recommendations to Medicare that will have broad implications for older adults with prediabetes (63). Another major strength is our ability to recruit a diverse study population from broad socioeconomic backgrounds, which will help us to better understand how implementation may need to be tailored for different populations and settings. Our team has the breadth and depth of experience in conducting behavioral intervention trials and implementation to analyze results and interpret them for stakeholders to inform potential future adoption of the DPP by a large healthcare system. We have also identified several challenges and discussed ways to address these. First, trials often have challenges recruiting people from underrepresented groups. Our experience recruiting underserved populations from health care systems enhances our ability to meet recruitment targets. Second, there is inherent measurement error associated with self-reported dietary intake, but we do not expect the measurement error to differ between randomized groups, allowing for a valid, randomized comparison. Third, though using technology to engage participants can be challenging, technology use among older adults is increasing (64), and our prior and ongoing work suggests it is feasible and increases opportunities for broader dissemination of successful interventions.

This work will generate a high fidelity, easily implemented adaptation of a theory-based DPP for older adults in a virtual setting. This will increase access to an evidence-based prediabetes intervention tailored to older adults, providing opportunities to address multiple potential barriers for care (e.g., travel, need for care partners, social distancing due to COVID-19). We anticipate that including virtual social support will increase adherence and maintain effectiveness of the intervention. The information gained will inform best practices for other virtual health interventions both for the general population as well as for older adults.

Online/remote learning is rapidly becoming a critical piece of the new normal going forward for DPP programs. Findings will inform policy decisions regarding the use of virtual DPP programs to prevent diabetes among the Medicare population to improve best practices in the delivery of an evidence-based intervention, having the potential to help over 26.4 million people with prediabetes (1).

## Author contributions

JB wrote the protocol, secured funding, and drafted the manuscript. EJ is a co-facilitator of the intervention sessions and contributed substantively to the development of the manuscript. MS provided expertise related to recruitment, retention, and group facilitation. MJ provided clinical insight to sample characteristics and interpretation of survey data. ER provided oversight of aim 2 measures and interpretation of pilot data to inform updating of implementation measures. HZ provided statistical guidance including power analyses and analytic plan. SZ and EG served as physician champions, recruited other physicians, and provided resources for intervention delivery. JC provided oversight of recruitment and retention and provided edits to the manuscript. All authors contributed to the article and approved the submitted version.

## Funding

Research reported in this publication was supported by the National Institute of Diabetes and Digestive and Kidney Diseases of the National Institutes of Health under award number R01 DK127916.

## Conflict of interest

The authors declare that the research was conducted in the absence of any commercial or financial relationships that could be construed as a potential conflict of interest.

## Publisher’s note

All claims expressed in this article are solely those of the authors and do not necessarily represent those of their affiliated organizations, or those of the publisher, the editors and the reviewers. Any product that may be evaluated in this article, or claim that may be made by its manufacturer, is not guaranteed or endorsed by the publisher.
