# Supplementary material for: Study protocol: BRInging the Diabetes prevention program to GEriatric Populations
Source: Front Med (Lausanne). 2023 May 18;10:1144156. doi: 10.3389/fmed.2023.1144156 (PMC10232977; doi:10.3389/fmed.2023.1144156)
Supplement: Supplementary file 1 [file Data_Sheet_1.docx]

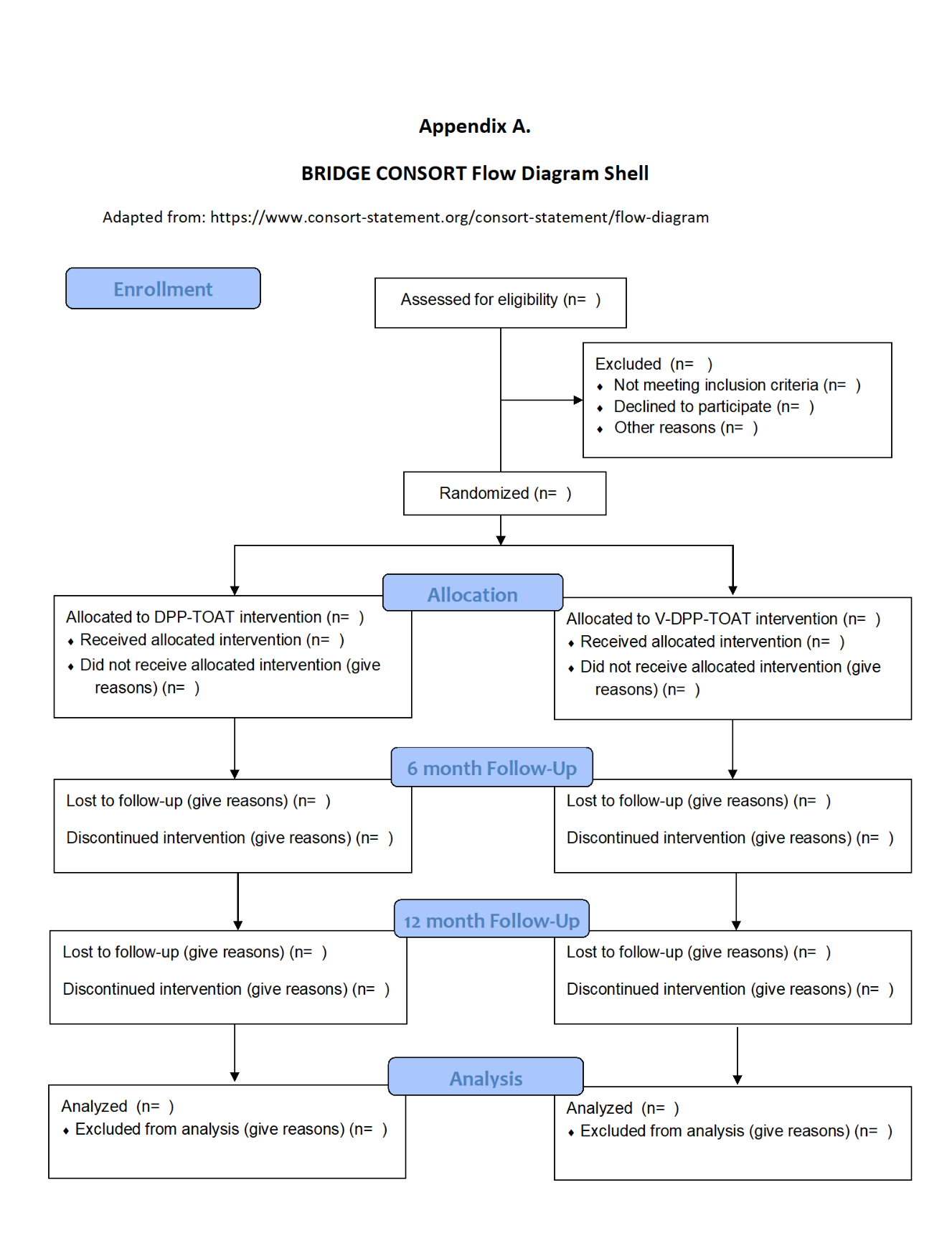


Appendix B: BRIDGE Intervention Session Topics

| Session 1 | Introduction to the Program |
| --- | --- |
| Session 2 | Get Active to Prevent Type 2 |
| Session 3 | Track Your Activity |
| Session 4 | Eat Well To Prevent Type 2 |
| Session 5 | Track Your Food |
| Session 6 | Get More Active |
| Session 7 | Energy In, Energy Out |
| Session 8 | Eating to Support Your Health Goals |
| Session 9 | Manage Stress |
| Session 10 | Eat Well Away From Home |
| Session 11 | Managing Triggers |
| Session 12 | Stay Active to Prevent Type 2 |
| Session 13 | Take Charge of Your Thoughts |
| Session 14 | Get Back on Track |
| Session 15 | Get Support |
| Session 16 | Stay Motivated To Prevent Type 2 |
| Session 17 | When Weight Loss Stalls |
| Session 18 | Take a Movement Break |
| Session 19 | Keep Your Heart Healthy |
| Session 20 | Shop and Cook to Prevent Type 2 |
| Session 21 | Get Enough Sleep |
| Session 22 | Prevent Type 2 for Life! |

*Sessions offered as part of the BRIDGE DPP; 22 of 26 possible sessions of the 2021 edition of the CDC’s DPP. https://www.cdc.gov/diabetes/prevention/resources/curriculum.html.*
